# Supplementary material for: Glial cell toxicity in a Drosophila C9orf72 neurodegeneration model
Source: PLoS Genet. 2026 Aug 5;22(8):e1011909. doi: 10.1371/journal.pgen.1011909 (PMC13480480; doi:10.1371/journal.pgen.1011909)
Supplement: Supplementary file 1 [file pgen.1011909.s008.docx]

**Response to reviewers for manuscript PGENETICS-D-25-01100**

We thank all three reviewers for their thoughtful and constructive critiques. We have fully revised the manuscript text and also have included results from several new experiments to address each of these critiques. Below is a point by point accounting for how we have responded to each critique:

**Reviewer #1 comment:** Dubnau identified an interesting role of glia in neurodegeneration in fly models of C9ALS/FTD. Interestingly, blocking apoptosis promotes, rather than suppresses neural defects in these models. Overall, this is an interesting paper with solid data.

**AU Response:** we are pleased that the reviewer felt this was an interesting and overall solid manuscript.

**Reviewer #1 comment:** My major concerns are about the relevance to disease and translation. Given the huge differences between human and fly glial system, it's unclear how these findings can be implicated in diseases. For example, the spg glia are very different in shape compared to oligodendrocytes, even though both construct the blood brain barrier. The spgs do not have the myolin sheath, which is disrupted in ALS/FTD.

**Au Response:** We of course agree that there are many differences between the fly and mammalian nervous systems. On the other hand, the basic signaling mechanisms that are at play within glial cells, between glia and neurons and within neurons are highly conserved. Fly genetic models of disease have been quite valuable for identification of underlying mechanisms. In the context of neurodegenerative disease, for example, fly models have provided evidence for nuclear/cytoplasmic signaling defects, defective autophagy, impact of DNA damage signaling and activation of retrotransposons such as LINE-like and endogenous retroviruses. In this specific case, the SPG are probably more similar to the endothelial cells that form the blood-brain barrier in mammals than to oligodendrocytes. The fly SPG and PNG come from a glial lineage, but then undergo a mesenchymal to endothelial transition to form a two layered barrier. They also likely retain some glial functions as well. We have revised the manuscript to make this more clear by the addition of this sentence to the introduction: “*The SPG are a specialized glial subtype that undergo a mesenchymal to endothelial transition to form a blood brain barrier*.”

**Reviewer #1 comment:** Furthermore, even though I agree with the cell non-autonomous role, I don't think their p35 data support such a role, as it can be simply explained as other cell-death pathways contribute to neuronal loss in their flies (e.g., necrosis, ferroptosis, etc.?).

**Au Response:** We appreciate this comment. Indeed, in response to other reviewer comments, we have revisited the non-cell autonomous effects and have added new data with better image quality and improved quantification across several additional timepoints. Taken together with the P35 and reaper effects, the data suggest that the non-cell autonomous effects do not cause neuronal loss, but do have some systemic effect that impacts lifespan separate from the effects of just loss of the glial cells. We actually agree with reviewer 1’s point that when we block apoptosis, the effects could be do to pushing the SPG into a different type of cell death and or senescence. Our revised manuscript includes this sentence in the discussion: “*Although we do not fully understand the underlying mechanisms, our findings are consistent with the idea that glial expression of G4C2 and GR can lead to several different cellular outcomes. This may include more than one possible path towards programmed cell death and/or cellular senescence (58–60). The choice of cell fate likely impacts severity and mechanisms of non-autonomous effects.*”

**Reviewer #1 comment:** In addition, I don't quite understand the mdg4-TDP43 part. Can the authors explain its role in ALS more clearly.

**Au Response:** We apologize for the lack of clarity. Our revised manuscript now includes this edited version of a section of the results: **“***We have previously reported that TDP-43 proteinopathy is associated with elevated expression of retrotransposons (RTEs) and endogenous retroviruses (ERVs), including the fly ERV mdg4 (29). Additionally, we previously reported that this ERV contributes to both cell-intrinsic toxicity as well as to the non-cell autonomous toxicity from glia to neurons in the Drosophila TDP-43 pathology model (26,27). These findings are convergent with results examining RTE expression in a TDP-43 model in mouse (42) and in postmortem cortical tissue from human subjects (30–33,43). Because C9orf72 patients exhibit TDP-43 pathology, we thus tested whether mdg4-ERV replication might also be induced in the presence of these C9orf72 transgenes*.”

**Reviewer #1 comment:** Nevertheless, I think this is a good fly paper with solid science.

**Au Response:** Thanks!

**Reviewer #2 comment:** This is a systematic study of toxicity arising from expression of several C9orf72 models (expanded G4C2 repeats, RNA-only models, and alternative codon “DPR only” models) in Drosophila glia. This complements existing research in C9orf72 toxicity and TDP43- associated toxicity including several studies investigating toxicity emanating from astrocyte expression in cell model systems.

The authors demonstrate:
• Reduced lifespan with pan-glial expression
• Reduced lifespan when expression is restricted to sub-pial glia
• De-repression of the mdg4 endogenous retrovirus
• Differences in severity not explained by co-expressing RNA-only and pure DPR models suggesting some other mechanism is at play.
• Anti-apoptotic manipulations (overexpression of p35) exacerbated non-cell autonomous effects of C9 glia while pro-apoptotic manipulations (overexpression of reaper) improved these effects.

Overall, the experiments in this study are well done. While it is largely descriptive and no new mechanisms are provided, it still adds some useful data to the field. There is prior published work of non-cell autonomous effects from glia with pathologic C9orf72 G4C2 expansions and this group did previously do similar studies with TDP-43 overexpression. However, this study is unique in the systematic use of several models (a “full” producing RNA and DPRs, a “no RNA”, and a “pure DPR” for poly GA, GR, PR, and PA.

**AU Response:** We appreciate the reviewer’s outline of the strengths of our manuscript.

**Reviewer 2 comment:**

The manuscript does have some shortcomings that should be addressed.

1. The abstract would benefit from a final sentence which summarizes the central conclusion(s) of the manuscript.

Au Response: Thanks for this suggestion. We have added this sentence to the end of the abstract: “*Together, these results indicate that expression of toxic C9orf72 components in glia produces deleterious effects on lifespan, though potentially through different mechanisms than seen in TDP-43 models of ALS/FTD*.”

**Reviewer 2 comment:**
2. The study is limited to male flies. Given that sexual dimorphism causes female flies to often exhibit different survival patterns compared to males, at least some comparison between female and male flies for key experiment al findings would strengthen the manuscript.

**Au Response:** Good suggestion. We have added a new supplemental figure (S1) that shows lifespans of female flies with each of the transgenes. The results are not meaningfully different from that seen with males.

**Reviewer 2 comment:**
3. The tools used are a bit heavy handed, which limits interpretation. For example, killing the cells in which you are trying to express a toxic factor and then having that prevent the toxicity is a bit of a circular argument. It is impressive that getting rid of glia alone (via reaper expression) is so well tolerated, but that doesn’t make the results any less tautological. The opposite studies (suppressing apoptosis with p35) are confounded because cells dying by a means other than apoptosis may just release factors that are problematic for the neighboring neurons in a fashion that is non-specific. All these issues could be addressed by expressing or treating with something that kills the glia by a fashion other than apoptosis or ALS. If not, some rewording of their interpretation in the context of these limitations is warranted

**Au Response:** We agree with part of what the reviewer is saying. In the case of the reaper over-expression, we are directly driving the apoptotic signaling pathway while also over-expressing the GR DPR or the G4C2 repeat expansion. The rationale for this experiment is to test whether the effects on lifespan from GR and G4C2 could be explained purely by the loss of the glial cells (just a subset of glia, the SPG). If so, then adding the reaper transgene should if anything make the animals die more quickly by killing the glia more quickly. Instead, when we use rpr to ablate the SPG subset of glia, it actually extends lifespan of the animals that co-express GR or G4C2 within those SPG. To our minds, this is a pretty striking result. With P35 expression, we are doing the opposite. We are blocking the apoptotic pathway in the SPG, and this causes the GR or G4C2 expressing flies to have shorter lifespans. This is consistent with our interpretation that the SPG are releasing something toxic, and that keeping them alive allows more of that toxic factor to be released. But the reviewer makes an astute point that this also is consistent with the possibility that when we block apoptosis, we drive these SPG into some other type of cell death (or senescence), leading to release of a toxic factor. Our manuscript ends with this text:

“*Although we do not fully understand the underlying mechanisms, our findings are consistent with the idea that glial expression of G4C2 and GR can lead to several different cellular outcomes. This may include more than one possible path towards programmed cell death and/or cellular senescence (58–60). The choice of cell fate likely impacts severity and mechanisms of non-autonomous effects*.”

We hope the reviewer will agree that this addresses the critique.

**Reviewer 2 comment:**
4. I struggle with their over-interpretation of their findings. For example, while they imply/suggest that there is a connection between the non-cell autonomous toxicity observed in C9orf72 models and TDP-43/TBPH, they do not show this despite feasible experiments that could be done to delineate the relationship (e.g. assess for glial TBPH cytoplasmic localization, as was done here for fly neurons: Lee et al 2024, [https://protect.checkpoint.com/v2/r01/___https://doi.org/10.1016/j.mocell.2023.12.003___.YzJ1OnN0b255YnJvb2s6YzpnOjQwZjkzZjQ4ZGNmOWM1MTg2NDZlNzFhZTE3OTMwNDFlOjc6ZjA4NDpmYzk3NTUxY2QyMWU5NmVjYTQxNGRhODk0ZWJiOTRkYWEyMWEyOTQ1Y2JmMmQ1YjliY2JlNDY2MjY5MWZlNTAwOnQ6VDpG](https://protect.checkpoint.com/v2/r01/___https:/doi.org/10.1016/j.mocell.2023.12.003___.YzJ1OnN0b255YnJvb2s6YzpnOjQwZjkzZjQ4ZGNmOWM1MTg2NDZlNzFhZTE3OTMwNDFlOjc6ZjA4NDpmYzk3NTUxY2QyMWU5NmVjYTQxNGRhODk0ZWJiOTRkYWEyMWEyOTQ1Y2JmMmQ1YjliY2JlNDY2MjY5MWZlNTAwOnQ6VDpG); Overexpress a form of TDP43 lacking a nuclear export signal in these same glia to see if you can suppress the repeat phenotype). Thus, they should either demonstrate a direct connection (something that would have real value to the field and would significantly enhance your manuscript) or they should not speculate based on the limited data they present.

**Au Response:** We thank the reviewer for these comments, which we agree with. We agree that we have not presented evidence that the toxicity of the C9orf72 transgenes are mediated by effects on TDP-43. In our hands, the available TBPH antibodies do not work well enough to detect nuclear clearance and cytoplasmic accumulation. So we have not been able to directly test whether the GR or G4C2 transgenes cause TDP-43 pathology. We did not intend to imply that we have shown this connection to be true, so if the reviewer took that interpretation from our text then we did not effectively communicate. Rather, our intent was only to point to the fact that in the literature, there is evidence that patients with C9orf72 repeat expansions also exhibit TDP-43 pathology. This was the motivation for us to examine effects on ERV activation, which is a hallmark of TDP-43 pathology, and to examine non cell autonomous effects, which also are hallmarks of TDP-43 pathology. In fact, what we observe is somewhat different from what we see with TDP-43 pathology in this same animal model. We have revised the text to include this explanation as the motivation for looking at non-cell autonomous effects and at mdg4 (hallmarks of TDP-43 pathology):

“*Although it is not clear whether C9orf72 repeat transgene expression in glia causes protein pathology of TBPH (the fly TDP-43 ortholog), the presence of TDP-43 pathology in C9orf72 patients (35) prompted us to examine whether expression of individual components of the C9orf72 repeat expansion in glia might recapitulate some of the toxic mechanisms that we see with glial overexpression of TDP-43*.”
We hope the reviewer agrees with and accepts this revision.

**Reviewer 2 comment:**
5. Similarly, I struggle with interpretation of the mdg4 story. It appears to be de-repressed more in some contexts but not others but not always in perfect correlation with phenotypes. They interpret this as evidence that it is not a cause of the phenotype. The right experiment to assess for a direct role for mdg4 would be expression of mdg4 in isolation in glial cells and see if that recapitulates the phenotype. To test the opposite, you could express an siRNA against mdg4 or knock it out and see if the repeat/DPR effects are mitigated. If mdg4 is really a marker of an ongoing process rather than a driver, then you could target those processes more directly. Without these more focused studies, I think their discussion needs to be amended.

**Au Response**: Our motivation for examination of mdg4 is that ERV expression is a hallmark of disease across a variety of TDP-43 models, and is seen in many cases of both sporadic ALS and C9orf72. Because we have seen activation of this fly ERV in our TDP-43 models, we examined whether this also was true in this context. We agree with the reviewer that in this study we use this mdg4-ERV reporter as a marker and do not investigate its functional role, and we have attempted to make that more clear. We found that the mdg4-reporter was activated in the presence of the GR and the G4C2 transgenes, which are most toxic. But it was not activated in the presence of the RNA only transgene or the GA DPR transgene, which are less toxic. This is consistent with the interpretation that mdg4 is a marker of toxicity. But the reviewer is correct that our manuscript previously was a bit unclear about whether or not the mdg4 correlated well with the other effects seen. This is because we had previously reported a difference between the effects of SPG expression of the GR and G4C2 transgenes on loss of neurons. But in response to a comment from reviewer 3 (see next section), we have done a more thorough and experimentally higher quality quantification of the numbers of neurons. We find that with SPG expression of either the GR or G4C2 transgene does not cause loss of neurons. Yet both of these transgenes exhibit equivalent effects with co-expression of reaper and P35. This helps to clarify that the mdg4 reporter correlates well with the cell intrinsic toxicity level of the four C90rf72 transgenes. We have revised this section of the results to read as follows:

“*Using the CLEVR reporter, we quantified the number of SPG that exhibited mdg4 replication after 6 days of expression of each of the C9orf72 transgenes. We found that expression of either (G4C2)36 or GR36 transgenes caused significant increases in the number of SPG that exhibit mdg4 replication events, with somewhat higher numbers being observed in the GR36 group (Figure 3A, B). By contrast, expression of the (G4C2)36 RO and GA36 transgenes had no significant effects on mdg4 replication (Figure S5). Thus the two transgenes that exhibit robust toxicity when expressed in SPG glia also exhibit robust effects on mdg4-ERV. This suggests that ERV activation is a marker of C9orf72 mediated toxicity as is the case for TDP-43. The fact that both (G4C2)36 and GR36 caused mdg4 replication is intriguing given that these two transgenes are significantly more toxic than the RO or GA36. But we also were struck by the fact that neither the (G4C2)36 nor GR36 produced measurable effects on survival of nearby neurons. This is in contrast with the case of TDP-43, where over-expression in SPG also causes induction of mdg4, and where mdg4 contributes to the non-cell autonomous effects on neuronal survival (Chang, 2019). We therefore wondered whether glial expression of (G4C2)36 or GR36 might trigger non-cell autonomous effects that we were unable to detect by quantifying neuronal cell loss.”*

We hope that the reviewer finds this response acceptable.

**Reviewer #3 comment:**

C9ORF72 G4C2 repeat expansion is the most common genetic cause of both ALS and FTD. The repeat expansion is bidirectionally transcribed into sense and anti-sense RNA foci, and also produces dipeptide repeats (DPRs) via a non-canonical translation mechanism. In this manuscript, the authors used glial cell type-specific expression of G4C2 repeats, or individual DPRs, or RNA repeat-only to systematically investigate both the glial cell-intrinsic and non-cell autonomous toxicity. They show that GR and G4C2 repeats produce the highest toxicity in glia. Only G4C2 repeat transgene produces non-cell autonomous toxicity that result in loss of nearby neurons. It was hypothesized that inhibiting glial cell death would increase neuronal toxicity. However, blocking apoptosis in GR- or G4C2-expressing glia using the p35 caspase inhibitor neither prevented glial death nor increased neuronal loss, yet still exacerbated the reduction in lifespan. Although this observation is intriguing, the underlying mechanism remains unclear.

**Au Response:** We thank the reviewer for this summary, which we fully agree with.

**Reviewer** **#3 comment**:
1. Translation in the GA frame is most efficient among the GP, GA, and GR frames on G4C2 repeats, due to the presence of a near-cognate start codon. However, the data in this manuscript show substantially higher levels of GR, which is inconsistent with prior studies. The sequence of the G4C2 transgene therefore needs to be clarified, particularly the region upstream of the repeats. If this sequence does not reflect the authentic C9orf72 context, the relative toxicity observed from the G4C2 transgene compared with DPR-only or repeat-only constructs may not accurately represent endogenous mechanisms. The conclusions should be interpreted more cautiously, and a more careful discussion is needed.

**Au Response:** This is an astute point from the reviewer. These transgenes were produced by Mizielinska et al (2014) using recursive directional ligation of fragments that contained 3 or 4 copies of a GGGGCC repeat. So the G4C2 transgene used in our manuscript does not include a near cognate start codon upstream of the GA DPR. So we agree with the reviewer that the relative levels of the three sense DPRs may not reflect what is seen in human subjects. On the other hand, the higher levels of expression of GR than GA with these transgenes are consistent with prior reports. And more importantly, the separate DPR transgenes that we used drive AUG-mediated translation of each of the DPRs, supporting the conclusion that GR is the most toxic. We have clarified this in the discussion section with the addition of this comment:

“*It is worth note that the G4C2 repeat used in this study does not contain the near cognate AUG that is present in the human C9orf72 repeat, which may drive higher levels of GA compared to other sense DPRs. So it is possible that the higher levels of GR produced by this transgene may differ from the situation in human disease. On the other hand, the individual DPR transgenes confirm that this arginine rich repeat is the most toxic in glia, as is reported in neurons as well*.”

We thank the reviewer for pointing this out.

**Reviewer #3 comment:**
2. In Figure 2B, the overall signal in the G4C2 and GR groups appears markedly dimmer, which is inconsistent with the quantification shown in Figure 2C. If there is an approximately 50% reduction in SPG cell number, one would expect a loss of signal in roughly half of the cells, with the remaining cells exhibiting comparable intensity to controls, rather than a uniform decrease in signal intensity across all cells.

**Au Response:** On this point we do not agree with the reviewer. Fig.2B shows quantification of the SPG glia, which are marked by expression of a UAS-nuclear-mCherry reporter under the SPG-Gal4 driver line. This marks each SPG nucleus so that we can count the total number of such nuclei. In the animals that express G4C2 or GR in these SPG, there are fewer SPG remaining. And those that are still remaining in the brain do express lower levels of the reporter, probably because they are already pretty sick cells that will soon die or be removed from the brain. So in this case, the less intense labeling in these genotypes is the result itself, it is evidence that G4C2 and GR are toxic to the SPG. And this is consistent with the quantification, which shows fewer SPG detected. We do agree that it is possible that there are some SPG that are still present in the brain, but they are so dysfunctional that they mCherry signal has weakened to the point that we cannot detect it. This caveat has been added to the results section, with the addition of the following comment:

“*One caveat of interpretation is that we cannot rule out the possibility that in the presence of GR or G4C2 expression, the SPG become so sick or dysfunctional that they cease to express the mCherry reporter.*”

We hope the reviewer will accept this explanation and revision to the text.

**Reviewer #3 comment:**
3. In Figure 3E, the overall image quality is low and shows issues similar to those noted in Figure 2B. Both GA and GR conditions also exhibit reduced signal intensity, and the staining in the G4C2 condition seems not work. These technical concerns make it difficult to interpret the data.

**Au Response:** On this point, we totally agree with the reviewer. The overall quality of the neuronal (elav) labeling in Fig.2E was quite poor (The reviewer said 3E, but we assume they are referring to 2E. There is no 3E), which made quantification of the number of neurons difficult. This was not due to a technical failure of the tissue processing or imaging. Rather, the reason for this is that in this experiment, we had also co-expressed a fluorescent reporter to mark the SPG glia and also used a Repo-antibody to mark all glia. This pushed the fluorescent detection of the neuronal Elav marker into the 405nm range, which in our hands is the most problematic secondary antibody. We had done it this way so that we could quantify the loss of SPG glia in the same experiment. To address this concern, we repeated this experiment without expressing a fluorescent reporter in the SPG. This freed up a channel on the confocal, and enabled us to pair a better secondary antibody with the Elav primary for detection of neuronal nuclei. Indeed, this has greatly improved the quality of the neuronal label, and made our quantification more robust.

With this higher quality neuronal labeling, we find that neither the GR nor G4C2 transgenes reduce the number of neurons when they are expressed in glial cells. This also leads to a more clear interpretation of the findings overall: GR and G4C2 are the most toxic transgenes when expressed in glia, they have equivalent effects on cell intrinsic toxicity and on mdg4-ERV activation. Neither transgene causes neuronal death when expressed in neurons. Both transgenes behave equivalently in combination with rpr and P35 .

We are indebted to reviewer 3 for this critique, which has greatly improved the manuscript.

**Reviewer #3 comment:**
4. Figure 4, transgene expression levels should be measured to confirm the expression is comparable in each group.

**Au Response:** The levels of expression of each transgene (quantified with Q-RT-PCR) were reported in supplemental Figure S2A rather than in Fig.4. The one transgene whose transcript levels accumulate to a somewhat higher level is the RNA only transgene, which does not impact figure 4 or correlate with toxicity levels (since RNA only is the least toxic).

**Reviewer #3 comment:**
5. Apoptosis markers should be stained for validate the apoptosis is inhibited for enhanced by the modifiers.

**Au Response:** We do not have good markers of apoptosis established to work in the adult brain. But rpr is commonly used to drive apoptosis in Drosophila and P35 is established as a caspase inhibitor that blocks apoptosis. We also have previously established that rpr expression ablates glial cells in the adult brain (Chang et al., 2019; Krupp et al., 2021) and that P35 expression prevents glial cell death in the context of TDP-43 pathology (Chang et al., 2019). But we agree with the thrust of this and the next critique of this reviewer and have edited the manuscript to be more circumspect in the conclusions that we draw (see response to next comment).

**Reviewer #3 comment:**
6. Blocking apoptosis in GR- or G4C2-expressing glia using the p35 caspase inhibitor neither prevented glial death nor increased neuronal loss, yet nevertheless exacerbated the reduction in lifespan. No mechanistic explanation is provided to account for this discrepancy. The manuscript claims that “these findings support the idea that in response to either (G4C2)36 or GR36 expression in SPG, cell-intrinsic effects that lead to apoptotic cell death have a protective role to the surrounding tissue and to the animal’s lifespan”. However, no evidence is presented to support an effect on surrounding tissues, and the basis for changes in lifespan remains unclear.

**Au response:** It is a fair critique that we do not have a mechanistic explanation for these findings. Our findings are that an established inhibitor of caspase 3 shortens lifespan of GR and G4C2 and an established proapoptotic gene extends lifespan of these two transgenes. And the apoptosis driver and inhibitor have the opposite impact on flies that do not express the GR or G4C2 transgenes. We do think that these themselves are quite provocative and worth reporting. But we agree that we should be far more circumspect in our conclusions. We have edited the discussion of these results to include a sentence that reads: “*Together, these results suggest the possibility that G_4_C_2_ and GR are capable of exerting some sort of non-cell autonomous effect that impacts lifespan even though it does not result in a measurable loss of neurons*.” And later in that same paragraph we now state: “*We do not understand the underlying mechanisms that explain this, but our findings are suggest the hypothesis that glial expression of G4C2 and GR can lead to several different cellular outcomes. This may include more than one possible path towards programmed cell death and/or cellular senescence (58–60). The choice of cell fate may impact severity and mechanisms of non-autonomous effects*.”

We hope that the reviewer finds our more circumspect discussion of these results to be acceptable.
