# Supplementary material for: Glial cell toxicity in a Drosophila C9orf72 neurodegeneration model
Source: PLoS Genet. 2026 Aug 5;22(8):e1011909. doi: 10.1371/journal.pgen.1011909 (PMC13480480; doi:10.1371/journal.pgen.1011909)
Supplement: Peer review history

# Peer review: Decision Letter 0

**DOI:** 10.1371/journal.pgen.1011909.r001

**Authors:** Anne O'Donnell-Luria, Louise Cheng

7 Jan 2026

PGENETICS-D-25-01100

Glial cell-intrinsic and non-cell autonomous toxicity in a Drosophila C9orf72 neurodegeneration model

PLOS Genetics

Dear author,

Thank you for submitting your manuscript to PLOS Genetics. After careful consideration, we feel that it has merit but does not fully meet PLOS Genetics's publication criteria as it currently stands. Therefore, we invite you to submit a revised version of the manuscript that addresses the points raised during the review process.

Please submit your revised manuscript in 2 months. If you will need more time than this to complete your revisions, please reply to this message or contact the journal office at plosgenetics@plos.org. When you're ready to submit your revision, log on to https://www.editorialmanager.com/pgenetics/ and select the 'Submissions Needing Revision' folder to locate your manuscript file.

Please include the following items when submitting your revised manuscript:

* A letter that responds to each point raised by the editor and reviewer(s). You should upload this letter as a separate file labeled 'Response to Reviewers'. This file does not need to include responses to any formatting updates and technical items listed in the 'Journal Requirements' section below.

* A marked-up copy of your manuscript that highlights changes made to the original version. You should upload this as a separate file labeled 'Revised Manuscript with Track Changes'.

* An unmarked version of your revised paper without tracked changes. You should upload this as a separate file labeled 'Manuscript'.

If you would like to make changes to your financial disclosure, competing interests statement, or data availability statement, please make these updates within the submission form at the time of resubmission. Guidelines for resubmitting your figure files are available below the reviewer comments at the end of this letter.

We look forward to receiving your revised manuscript.

Kind regards,

Louise Cheng

Academic Editor

PLOS Genetics

Anne O'Donnell-Luria

Section Editor

PLOS Genetics

Aimée Dudley

Editor-in-Chief

PLOS Genetics

Anne Goriely

Editor-in-Chief

PLOS Genetics

**Journal Requirements:**

1) Please ensure that the CRediT author contributions listed for every co-author are completed accurately and in full.

At this stage, the following Authors/Authors require contributions: Isabel Hubbard, and Josh Dubnau. Please ensure that the full contributions of each author are acknowledged in the "Add/Edit/Remove Authors" section of our submission form.

The list of CRediT author contributions may be found here: https://journals.plos.org/plosgenetics/s/authorship#loc-author-contributions

2) Please provide an Author Summary. This should appear in your manuscript between the Abstract (if applicable) and the Introduction, and should be 150-200 words long. The aim should be to make your findings accessible to a wide audience that includes both scientists and non-scientists. Sample summaries can be found on our website under Submission Guidelines:

https://journals.plos.org/plosgenetics/s/submission-guidelines#loc-parts-of-a-submission

3) Please upload all main figures as separate Figure files in .tif or .eps format. For more information about how to convert and format your figure files please see our guidelines:

https://journals.plos.org/plosgenetics/s/figures

4) We have noticed that you have uploaded Supporting Information files, but you have not included a list of legends. Please add a full list of legends for your Supporting Information files after the references list.

**Reviewers' comments:**

Reviewer's Responses to Questions

**Comments to the Authors:**

**Please note here if the review is uploaded as an attachment.**

Reviewer #1: Dubnau identified an interesting role of glia in neurodegeneration in fly models of C9ALS/FTD. Interestingly, blocking apoptosis promotes, rather than suppresses neural defects in these models. Overall, this is an interesting paper with solid data. My major concerns are about the relevance to disease and translation. Given the huge differences between human and fly glial system, it's unclear how these findings can be implicated in diseases. For example, the spg glia are very different in shape compared to oligodendrocytes, even though both construct the blood brain barrier. The spgs do not have the myolin sheath, which is disrupted in ALS/FTD. Furthermore, even though I agree with the cell non-autonomous role, I don't think their p35 data support such a role, as it can be simply explained as other cell-death pathways contribute to neuronal loss in their flies (e.g., necrosis, ferroptosis, etc.?). In addition, I don't quite understand the mdg4-TDP43 part. Can the authors explain its role in ALS more clearly.

Nevertheless, I think this is a good fly paper with solid science.

Reviewer #2: This is a systematic study of toxicity arising from expression of several C9orf72 models (expanded G4C2 repeats, RNA-only models, and alternative codon “DPR only” models) in Drosophila glia. This complements existing research in C9orf72 toxicity and TDP43- associated toxicity including several studies investigating toxicity emanating from astrocyte expression in cell model systems.

The authors demonstrate:

• Reduced lifespan with pan-glial expression

• Reduced lifespan when expression is restricted to sub-pial glia

• De-repression of the mdg4 endogenous retrovirus

• Differences in severity not explained by co-expressing RNA-only and pure DPR models suggesting some other mechanism is at play.

• Anti-apoptotic manipulations (overexpression of p35) exacerbated non-cell autonomous effects of C9 glia while pro-apoptotic manipulations (overexpression of reaper) improved these effects.

Overall, the experiments in this study are well done. While it is largely descriptive and no new mechanisms are provided, it still adds some useful data to the field. There is prior published work of non-cell autonomous effects from glia with pathologic C9orf72 G4C2 expansions and this group did previously do similar studies with TDP-43 overexpression. However, this study is unique in the systematic use of several models (a “full” producing RNA and DPRs, a “no RNA”, and a “pure DPR” for poly GA, GR, PR, and PA.

The manuscript does have some shortcomings that should be addressed.

1. The abstract would benefit from a final sentence which summarizes the central conclusion(s) of the manuscript.

2. The study is limited to male flies. Given that sexual dimorphism causes female flies to often exhibit different survival patterns compared to males, at least some comparison between female and male flies for key experiment al findings would strengthen the manuscript.

3. The tools used are a bit heavy handed, which limits interpretation. For example, killing the cells in which you are trying to express a toxic factor and then having that prevent the toxicity is a bit of a circular argument. It is impressive that getting rid of glia alone (via reaper expression) is so well tolerated, but that doesn’t make the results any less tautological. The opposite studies (suppressing apoptosis with p35) are confounded because cells dying by a means other than apoptosis may just release factors that are problematic for the neighboring neurons in a fashion that is non-specific. All these issues could be addressed by expressing or treating with something that kills the glia by a fashion other than apoptosis or ALS. If not, some rewording of their interpretation in the context of these limitations is warranted

4. I struggle with their over-interpretation of their findings. For example, while they imply/suggest that there is a connection between the non-cell autonomous toxicity observed in C9orf72 models and TDP-43/TBPH, they do not show this despite feasible experiments that could be done to delineate the relationship (e.g. assess for glial TBPH cytoplasmic localization, as was done here for fly neurons: Lee et al 2024, https://doi.org/10.1016/j.mocell.2023.12.003; Overexpress a form of TDP43 lacking a nuclear export signal in these same glia to see if you can suppress the repeat phenotype). Thus, they should either demonstrate a direct connection (something that would have real value to the field and would significantly enhance your manuscript) or they should not speculate based on the limited data they present.

5. Similarly, I struggle with interpretation of the mdg4 story. It appears to be de-repressed more in some contexts but not others but not always in perfect correlation with phenotypes. They interpret this as evidence that it is not a cause of the phenotype. The right experiment to assess for a direct role for mdg4 would be expression of mdg4 in isolation in glial cells and see if that recapitulates the phenotype. To test the opposite, you could express an siRNA against mdg4 or knock it out and see if the repeat/DPR effects are mitigated. If mdg4 is really a marker of an ongoing process rather than a driver, then you could target those processes more directly. Without these more focused studies, I think their discussion needs to be amended.

Reviewer #3: C9ORF72 G4C2 repeat expansion is the most common genetic cause of both ALS and FTD. The repeat expansion is bidirectionally transcribed into sense and anti-sense RNA foci, and also produces dipeptide repeats (DPRs) via a non-canonical translation mechanism. In this manuscript, the authors used glial cell type-specific expression of G4C2 repeats, or individual DPRs, or RNA repeat-only to systematically investigate both the glial cell-intrinsic and non-cell autonomous toxicity. They show that GR and G4C2 repeats produce the highest toxicity in glia. Only G4C2 repeat transgene produces non-cell autonomous toxicity that result in loss of nearby neurons. It was hypothesized that inhibiting glial cell death would increase neuronal toxicity. However, blocking apoptosis in GR- or G4C2-expressing glia using the p35 caspase inhibitor neither prevented glial death nor increased neuronal loss, yet still exacerbated the reduction in lifespan. Although this observation is intriguing, the underlying mechanism remains unclear.

1. Translation in the GA frame is most efficient among the GP, GA, and GR frames on G4C2 repeats, due to the presence of a near-cognate start codon. However, the data in this manuscript show substantially higher levels of GR, which is inconsistent with prior studies. The sequence of the G4C2 transgene therefore needs to be clarified, particularly the region upstream of the repeats. If this sequence does not reflect the authentic C9orf72 context, the relative toxicity observed from the G4C2 transgene compared with DPR-only or repeat-only constructs may not accurately represent endogenous mechanisms. The conclusions should be interpreted more cautiously, and a more careful discussion is needed.

2. In Figure 2B, the overall signal in the G4C2 and GR groups appears markedly dimmer, which is inconsistent with the quantification shown in Figure 2C. If there is an approximately 50% reduction in SPG cell number, one would expect a loss of signal in roughly half of the cells, with the remaining cells exhibiting comparable intensity to controls, rather than a uniform decrease in signal intensity across all cells.

3. In Figure 3E, the overall image quality is low and shows issues similar to those noted in Figure 2B. Both GA and GR conditions also exhibit reduced signal intensity, and the staining in the G4C2 condition seems not work. These technical concerns make it difficult to interpret the data.

4. Figure 4, transgene expression levels should be measured to confirm the expression is comparable in each group.

5. Apoptosis markers should be stained for validate the apoptosis is inhibited for enhanced by the modifiers.

6. Blocking apoptosis in GR- or G4C2-expressing glia using the p35 caspase inhibitor neither prevented glial death nor increased neuronal loss, yet nevertheless exacerbated the reduction in lifespan. No mechanistic explanation is provided to account for this discrepancy. The manuscript claims that “these findings support the idea that in response to either (G4C2)36 or GR36 expression in SPG, cell-intrinsic effects that lead to apoptotic cell death have a protective role to the surrounding tissue and to the animal’s lifespan”. However, no evidence is presented to support an effect on surrounding tissues, and the basis for changes in lifespan remains unclear.

**********

**Have all data underlying the figures and results presented in the manuscript been provided?**

Large-scale datasets should be made available via a public repository as described in the *PLOS Genetics*  data availability policy, and numerical data that underlies graphs or summary statistics should be provided in spreadsheet form as supporting information.

Reviewer #1: Yes

Reviewer #2: Yes

Reviewer #3: **No:**

**********

PLOS authors have the option to publish the peer review history of their article (what does this mean?). If published, this will include your full peer review and any attached files.

If you choose “no”, your identity will remain anonymous but your review may still be made public.

**Do you want your identity to be public for this peer review?**  For information about this choice, including consent withdrawal, please see our Privacy Policy.

Reviewer #1: **Yes:**  Ke Zhang

Reviewer #2: No

Reviewer #3: No

[NOTE: If reviewer comments were submitted as an attachment file, they will be attached to this email and accessible via the submission site. Please log into your account, locate the manuscript record, and check for the action link "View Attachments". If this link does not appear, there are no attachment files.]

**Figure resubmission:**

**Reproducibility:**

To enhance the reproducibility of your results, we recommend that authors of applicable studies deposit laboratory protocols in protocols.io, where a protocol can be assigned its own identifier (DOI) such that it can be cited independently in the future. Additionally, PLOS ONE offers an option to publish peer-reviewed clinical study protocols. Read more information on sharing protocols at https://plos.org/protocols?utm_medium=editorial-email&utm_source=authorletters&utm_campaign=protocols

# Author comment: Author response to Decision Letter 1

**DOI:** 10.1371/journal.pgen.1011909.r002

18 Jun 2026

Attachment Submitted filename: Response to reviewers for manuscript PGENETICS.docx

# Peer review: Decision Letter 1

**DOI:** 10.1371/journal.pgen.1011909.r003

**Authors:** Anne O'Donnell-Luria, Louise Cheng

22 Jul 2026

Dear Dr Dubnau,

We are pleased to inform you that your manuscript entitled "Glial cell toxicity in a Drosophila C9orf72 neurodegeneration model" has been editorially accepted for publication in PLOS Genetics. Congratulations!

Before your submission can be formally accepted and sent to production you will need to complete our formatting changes, which you will receive in a follow up email. This is also you chance to make any additional revisions related to the minor reviewer comments. Please be aware that it may take several days for you to receive this email; during this time no action is required by you. Please note: the accept date on your published article will reflect the date of this provisional acceptance, but your manuscript will not be scheduled for publication until the required changes have been made.

Once your paper is formally accepted, an uncorrected proof of your manuscript will be published online ahead of the final version, unless you’ve already opted out via the online submission form. If, for any reason, you do not want an earlier version of your manuscript published online or are unsure if you have already indicated as such, please let the journal staff know immediately at plosgenetics@plos.org.

In the meantime, please log into Editorial Manager at https://www.editorialmanager.com/pgenetics/, click the "Update My Information" link at the top of the page, and update your user information to ensure an efficient production and billing process. Note that PLOS requires an ORCID iD for all corresponding authors. Therefore, please ensure that you have an ORCID iD and that it is validated in Editorial Manager. To do this, go to ‘Update my Information’ (in the upper left-hand corner of the main menu), and click on the Fetch/Validate link next to the ORCID field.  This will take you to the ORCID site and allow you to create a new iD or authenticate a pre-existing iD in Editorial Manager.

If you have a press-related query, or would like to know about making your underlying data available (as you will be aware, this is required for publication), please see the end of this email. If your institution or institutions have a press office, please notify them about your upcoming article at this point, to enable them to help maximise its impact. Inform journal staff as soon as possible if you are preparing a press release for your article and need a publication date.

Thank you again for supporting open-access publishing; we are looking forward to publishing your work in PLOS Genetics!

Yours sincerely,

Louise Y. Cheng

Academic Editor

PLOS Genetics

Anne O'Donnell-Luria

Section Editor

PLOS Genetics

Aimée Dudley

Editor-in-Chief

PLOS Genetics

Anne Goriely

Editor-in-Chief

PLOS Genetics

www.plosgenetics.org

BlueSky: @plos.bsky.social

----------------------------------------------------

Comments from the reviewers (if applicable):

Reviewer's Responses to Questions

**Comments to the Authors:**

**Please note here if the review is uploaded as an attachment.**

Reviewer #1: The authors addressed my comments. I have two more suggestions:

1) Define G4C2

2) Put, in the Discussion, the authors' response on my concern about the relevance to disease and translation.

Reviewer #2: This is an interesting story regarding the role of SPG glia expressing GR DPR producing C9-relevant transgenes leading to cell non-autonomous toxicity independent of neuronal loss. This adds value to the C9 field and potentially to the intersection of neurodegeneration and aging. While the manuscript does not provide a clear mechanism by which this occurs, we do not feel that such is requisite to justify publication.

Reviewer #3: The manuscript has been strengthened by the addition of new data and careful revisions to improve clarity and avoid overstating the conclusions. Although the underlying molecular mechanism remains unclear, the findings are of sufficient interest to warrant publication, provided that the conclusions are presented cautiously and do not overstate the implications of the results.

**********

**Have all data underlying the figures and results presented in the manuscript been provided?**

Large-scale datasets should be made available via a public repository as described in the *PLOS Genetics*  data availability policy, and numerical data that underlies graphs or summary statistics should be provided in spreadsheet form as supporting information.

Reviewer #1: Yes

Reviewer #2: Yes

Reviewer #3: None

**********

PLOS authors have the option to publish the peer review history of their article (what does this mean?). If published, this will include your full peer review and any attached files.

If you choose “no”, your identity will remain anonymous but your review may still be made public.

**Do you want your identity to be public for this peer review?**  For information about this choice, including consent withdrawal, please see our Privacy Policy.

Reviewer #1: **Yes:**  Ke Zhang

Reviewer #2: **Yes:**  Peter Todd

Reviewer #3: No

----------------------------------------------------

**Data Deposition**

If you have submitted a Research Article or Front Matter that has associated data that are not suitable for deposition in a subject-specific public repository (such as GenBank or ArrayExpress), one way to make that data available is to deposit it in the Dryad Digital Repository. As you may recall, we ask all authors to agree to make data available; this is one way to achieve that. A full list of recommended repositories can be found on our website.

The following link will take you to the Dryad record for your article, so you won't have to re‐enter its bibliographic information, and can upload your files directly:

http://datadryad.org/submit?journalID=pgenetics&manu=PGENETICS-D-25-01100R1

More information about depositing data in Dryad is available at http://www.datadryad.org/depositing. If you experience any difficulties in submitting your data, please contact help@datadryad.org for support.

Additionally, please be aware that our data availability policy requires that all numerical data underlying display items are included with the submission, and you will need to provide this before we can formally accept your manuscript, if not already present.

----------------------------------------------------

**Press Queries**

If you or your institution will be preparing press materials for this manuscript, or if you need to know your paper's publication date for media purposes, please inform the journal staff as soon as possible so that your submission can be scheduled accordingly. Your manuscript will remain under a strict press embargo until the publication date and time. This means an early version of your manuscript will not be published ahead of your final version. PLOS Genetics may also choose to issue a press release for your article. If there's anything the journal should know or you'd like more information, please get in touch via plosgenetics@plos.org.

# Editor report: Acceptance letter

**DOI:** 10.1371/journal.pgen.1011909.r004

**Authors:** Anne O'Donnell-Luria, Louise Cheng

PGENETICS-D-25-01100R1

Glial cell toxicity in a Drosophila C9orf72 neurodegeneration model

Dear Dr Dubnau,

We are pleased to inform you that your manuscript entitled "Glial cell toxicity in a Drosophila C9orf72 neurodegeneration model" has been formally accepted for publication in PLOS Genetics! Your manuscript is now with our production department and you will be notified of the publication date in due course.

The corresponding author will soon be receiving a typeset proof for review, to ensure errors have not been introduced during production. Please review the PDF proof of your manuscript carefully, as this is the last chance to correct any errors. Please note that major changes, or those which affect the scientific understanding of the work, will likely cause delays to the publication date of your manuscript.

Soon after your final files are uploaded, unless you have opted out or your manuscript is a front-matter piece, the early version of your manuscript will be published online. The date of the early version will be your article's publication date. The final article will be published to the same URL, and all versions of the paper will be accessible to readers.

For Research Articles, you will receive an invoice from PLOS for your publication fee after your manuscript has reached the completed accept phase. If you receive an email requesting payment before acceptance or for any other service, this may be a phishing scheme. Learn how to identify phishing emails and protect your accounts at https://explore.plos.org/phishing.

Thank you again for supporting PLOS Genetics and open-access publishing. We are looking forward to publishing your work!

With kind regards,

Janani Seenivasan

PLOS Genetics

On behalf of:

The PLOS Genetics Team

Carlyle House, Carlyle Road, Cambridge CB4 3DN | United Kingdom

plosgenetics@plos.org | +44 (0) 1223-442823

plosgenetics.org | Twitter: @PLOSGenetics
